# Impact of the Pandemic on Psychiatric Research and Publications

**DOI:** 10.1192/j.eurpsy.2022.52

**Published:** 2022-09-01

**Authors:** P. Mohr

**Affiliations:** National Institute of Mental Health, Czech Republic, Nimh, Klecany, Czech Republic

## Abstract

In addition to the global negative impact on mental health of general population, as well as psychiatric patients, Covid-19 pandemic affected significantly research. Initially, lockdowns and restrictions of human contacts temporarily disrupted clinical research activities, but the unprecedented health crisis also provided unique opportunity to study epidemiology of mental disorders, direct and indirect effects of the pandemic on psychiatric disorders, underlying pathophysiological mechanisms, or long-term neuropsychiatric consequences. Research has refocused now on better understanding of the causes, presentations, outcome trajectories, and therapy of mental illnesses. New research topics are followed by the surge in publications covering Covid-19 and mental health and/or psychiatric disorders and treatments. The impact of the pandemic on research and publications is also evidenced by the results of a survey among psychiatric researchers.

**Disclosure:**

No significant relationships.

